# Focus On: Ethnicity and the Social and Health Harms From Drinking

**DOI:** 10.35946/arcr.v35.2.13

**Published:** 2014

**Authors:** Karen G. Chartier, Patrice A.C. Vaeth, Raul Caetano

**Affiliations:** **Karen G. Chartier, Ph.D.**, *is an instructor and assistant professor at the Virginia Commonwealth University School of Social Work and Department of Psychiatry with the Virginia Institute for Psychiatric and Behavioral Genetics, Richmond, Virginia.*; **Patrice A.C. Vaeth, Dr.P.H.**, *is a scientist at the Prevention Research Center, Pacific Institute for Research and Evaluation, Berkeley, California.*; **Raul Caetano, M.D., Ph.D.**, *is regional dean and professor at the University of Texas School of Public Health, Dallas Regional Campus, Dallas Texas.*

**Keywords:** Alcohol consumption, alcohol-attributable fractions, alcohol burden, harmful drinking, alcohol and other drug, induced risk, risk factors, ethnicity, ethnic groups, racial groups, cultural patterns of drinking, Native Americans, Hispanics, Blacks, African Americans, Asian Americans, Whites, Caucasians, injury, intentional injury, unintentional injury, fetal alcohol syndrome, gastrointestinal diseases, cardiovascular diseases, cancers, diabetes, infectious diseases

## Abstract

Alcohol consumption is differentially associated with social and health harms across U.S. ethnic groups. Native Americans, Hispanics, and Blacks are disadvantaged by alcohol-attributed harms compared with Whites and Asians. Ethnicities with higher rates of risky drinking experience higher rates of drinking harms. Other factors that could contribute to the different effects of alcohol by ethnicity are social disadvantage, acculturation, drink preferences, and alcohol metabolism. This article examines the relationship of ethnicity and drinking to (1) unintentional injuries, (2) intentional injuries, (3) fetal alcohol syndrome (FAS), (4) gastrointestinal diseases, (5) cardiovascular diseases, (6) cancers, (7) diabetes, and (8) infectious diseases. Reviewed evidence shows that Native Americans have a disproportionate risk for alcohol-related motor vehicle fatalities, suicides and violence, FAS, and liver disease mortality. Hispanics are at increased risk for alcohol-related motor vehicle fatalities, suicide, liver disease, and cirrhosis mortality; and Blacks have increased risk for alcohol-related relationship violence, FAS, heart disease, and some cancers. However, the scientific evidence is incomplete for each of these harms. More research is needed on the relationship of alcohol consumption to cancers, diabetes, and HIV/AIDS across ethnic groups. Studies also are needed to delineate the mechanisms that give rise to and sustain these disparities in order to inform prevention strategies.

Research has shown differential social and health effects from alcohol use across U.S. ethnic groups, including Whites, Blacks, Hispanics, Asians, and Native Americans. The relationship of ethnicity to alcohol-related social and health harms partially is attributed to the different rates and patterns of drinking across ethnicities. Some ethnic groups have higher rates of alcohol consumption, putting them at greater risk of drinking harms. However, other ethnic minorities experience health harms from drinking that are disproportionate to their consumption. Differences in social and socioeconomic factors and biological differences related to alcohol metabolism also could contribute to alcohol’s varying effects across populations. This article reviews current research examining the harms of drinking for U.S. ethnic groups. It examines such social harms as driving under the influence and alcohol-attributed violence but primarily focuses on health harms like fetal alcohol syndrome (FAS), liver diseases, and cancers.

The research reviewed focuses on Whites, Blacks, Hispanics, Asians, and Native Americans (i.e., American Indians and Alaska Natives) in the United States as general ethnic groups, although significant subgroup differences within populations also are evident. There are limitations to using these general categories because ethnicity encompasses a combination of characteristics such as tribe, ancestry, national group, birthplace, and language, which could have distinct relationships to patterns of drinking and alcohol-related harms (Caetano 1986; Cheung 1993; Heath 1990–1991). People with multiethnic backgrounds also are not well represented by these general groups. Nevertheless, studies that examine ethnicity and alcohol-attributed harms provide important information about public health and serve to identify high-risk groups in the population. This article shows that Native Americans, Hispanics, and Blacks are disproportionately affected by the adverse social and health harms from alcohol consumption.

## Drinking Patterns and Other Determinants of Risk for Alcohol-Related Harms

Heavy drinking and binge drinking contribute to a variety of alcohol-attributed social and health harms (Naimi et al. 2003; Rehm et al. 2010). Heavy alcohol use, as defined by the National Institute on Alcohol Abuse and Alcoholism’s (NIAAA’s) *Helping Patients Who Drink Too Much: A Clinician’s Guide* (NIAAA 2005), is defined as consuming more than 4 standard drinks per day (or more than 14 per week) for men and more than 3 per day (or more than 7 per week) for women. One standard drink is equivalent to 12 ounces of beer, 5 ounces of wine, or 1.5 ounces of 80-proof spirits. Binge drinking is defined as consuming five or more drinks in approximately 2 hours for men and four or more drinks for women (NIAAA 2004).

Other than these patterns of consumption, the volume of alcohol intake, defined as the total alcohol consumed over a time period, is linked to social and health harms. Most diseases (e.g., injury, some cancers, and liver cirrhosis) have a detrimental dose-response relationship with alcohol as risk increases with higher-volume alcohol consumption, whereas coronary heart disease and diabetes display a J- or U-shaped relationship (Howard et al. 2004; Rehm et al. 2010; Roerecke and Rehm 2012). The J and U shapes are characterized by both detrimental and beneficial (e.g., increased high-density lipoprotein “good cholesterol”) (Goldberg and Soleas 2001) effects of alcohol use, with higher risks for abstainers and heavy drinkers compared with light or moderate drinkers. However, this relationship is complex and varies by age, gender, and ethnicity (Roerecke and Rehm 2012). Drinking levels that may be protective of cardiovascular health among men also may increase the risk for other harms such as injury, violence, gastrointestinal disease, and some cancers.

Epidemiological studies show that these high-risk patterns of drinking and drinking volume vary by U.S. ethnic group. Ethnicities with greater drinking volume and higher rates of daily and weekly heavy drinking could be at greater risk for experiencing alcohol-attributed harms. Among adult drinkers in the United States, based on the 2001–2002 National Epidemiologic Survey on Alcohol and Related Conditions (NESARC) (Chen et al. 2006), Native Americans and Hispanics have greater alcohol consumption than other ethnic minority groups. Rates of daily heavy drinking were higher among Hispanics (33.9 percent), Native Americans (28.4 percent), and Whites (27.3 percent) compared with Blacks (22.5 percent) and Asians (19.2 percent). Weekly heavy drinking was highest among Native Americans (21.9 percent), followed by Blacks (16.4 percent), Whites (16.3 percent), Hispanics (11.8 percent), and Asians (9.8 percent). Based on the 2001–2002 NESARC data, Caetano and colleagues (2010) reported that White men consumed a higher volume of alcohol (22.3 drinks per month) than Black men (18.9 drinks per month) and Hispanic men (17.8 drinks per month) and that White women consumed more (6.2 drinks per month) compared with Black women (4.9 drinks per month) and Hispanic women (3.9 drinks per month). The sample for these estimates of drinking volume was the U.S. population of Whites, Blacks, and Hispanics and included abstainers. However, a study by Mulia and colleagues (2009) of current drinkers in the United States showed that Whites consumed less alcohol than Hispanics and more than Blacks. The differences between these two studies could reflect a higher rate of abstinence from alcohol among Hispanics (25.7 percent) compared with Whites (13.4 percent) in the U.S. population (Chen et al. 2006). The study that included abstainers (Caetano et al. 2010), who by definition consume zero drinks, showed higher drinking volume for Whites, whereas the study excluding abstainers (Mulia et al. 2009) reported higher volume for Hispanics. Other ethnic minority groups with higher abstinence rates include Blacks (24.7 percent) and Asians (39.1 percent). Native Americans (17.14 percent) have lower rates of abstinence than other minority groups.

Alternatively, the negative effects from drinking could be explained by factors other than alcohol consumption. Mulia and colleagues (2009) showed that Black and Hispanic adult drinkers were more likely than White drinkers to report alcohol dependence symptoms and social problems from drinking at the no/low level of heavy drinking. Blacks also experience negative health effects from alcohol use despite showing a later onset of use and levels of use often comparable with, if not lower than, Whites (Chartier et al. 2011; Chen et al. 2006; Russo et al. 2004). Other factors associated with ethnic disparities in alcohol-related harms include social disadvantage, characterized by lower socioeconomic status, neighborhood poverty, greater neighborhood alcohol availability, reduced alcohol treatment utilization, and unfair treatment or discrimination (Chae et al. 2008; Chartier and Caetano 2011; Cunradi et al. 2000; Mulia et al. 2008; Nielsen et al. 2005; Zemore et al. 2011). Some ethnic subgroups are more likely to consume high-alcohol-content beverages (e.g., malt liquor), which could result in greater social and health harms (Vilamovska et al. 2009). Preference for such beverages seems to be more common in lower-income ethnic minority communities (Bluthenthal et al. 2005). Some ethnic minority groups also face stressors related to the acculturation process. Higher acculturation, U.S.-born nativity, and longer residence in the United States are risk factors associated with alcohol use disorders and alcohol-related social problems among Hispanics, particularly women (Alegria et al. 2007, 2008; Caetano et al. 2009, 2012; Zemore 2007). Another potential contributor is ethnic differences in the alcohol content of poured drinks. Kerr and colleagues (2009) showed that Black men had drink sizes with larger average alcohol content compared with other groups, which partially could explain the higher risks for alcohol-related harms. Genes responsible for alcohol metabolism also vary across ethnic groups and could be associated with susceptibility for alcohol-related diseases. Among Whites, Blacks, and Asians, alcohol dehydrogenase (ADH) and aldehyde dehydrogenase (ALDH) genotypes have been linked in combination with drinking to alcohol-related cancers, birth defects, and pancreatitis (Yin and Agarwal 2001).

## Ethnicity and Alcohol-Attributed Harms

Alcohol-attributed harms can be both acute and chronic conditions that are wholly caused (e.g., alcoholic liver cirrhosis) or associated with alcohol use via intoxication, alcohol dependence, and the toxic effects of alcohol (Rehm et al. 2010). The major injury and disease categories linked to alcohol consumption include (1) unintentional injuries, (2) intentional injuries, (3) FAS, (4) gastrointestinal diseases, (5) cardiovascular diseases, (6) cancers, (7) diabetes, and (8) infectious diseases (World Health Organization [WHO] 2011). Evidence is incomplete on the relationship between ethnicity, drinking, and each of these categories. Below, those alcohol-related harms are described that have available findings by ethnic group in addition to important gaps in this scientific literature. Alcohol use disorders are causally linked to drinking and vary by ethnicity (i.e., more likely in Native Americans and Whites) (Hasin et al. 2007), but this disease category is not described here.

## Unintentional Injuries

Unintentional injuries associated with alcohol use include falls, drowning, and poisoning (WHO 2011). However, most available research on ethnicity, alcohol use, and injuries is focused on motor vehicle crashes. Alcohol-impaired driving and crash fatalities vary by ethnicity, with Native Americans and Hispanics being at higher risk than other ethnic minority groups. Past-year driving under the influence (DUI) estimates based on the 2007 National Survey on Drug Use and Health were highest for Whites (15.6 percent) and Native Americans (13.3 percent) relative to Blacks (10.0 percent), Hispanics (9.3 percent), and Asians (7.0 percent) (Substance Abuse and Mental Health Services Administration [SAMHSA] 2008). National surveys generally show lower DUI rates for Hispanics than Whites, but studies based on arrest data identify Hispanics as another high-risk group for DUI involvement (Caetano and McGrath 2005; SAMHSA 2005). The DUI arrest rate for Native Americans in 2001, according to the U.S. Department of Justice (Perry 2004), was 479 arrestees per 100,000 residents compared with 332 for all other U.S. ethnic groups.

Based on a 1999–2004 report from the National Highway Traffic Safety Administration (Hilton 2006), rates of intoxication (i.e., blood alcohol concentration [BAC] more than or equal to 0.08 percent) for drivers who were fatally injured in a motor vehicle crash were highest for Native Americans (57 percent) and Hispanics (47 percent) and lowest for Asians (approximately 20 percent), with Whites and Blacks falling in between. Across ethnic groups, most drinking drivers killed were male, although the proportion of female drivers who were intoxicated among fatally injured drivers was highest (i.e., more than 40 percent) for Native Americans. Centers for Disease Control and Prevention (CDC) (2009*b*) statistics on alcohol-related motor vehicle crash deaths also point to an important subgroup difference for Asians. In 2006, the overall death rate among Asians (1.8 per 100,000 people) obscured the death rate among Native Hawaiians and other Pacific Islanders (5.9), which was less than the rate for Native Americans but similar to that for Hispanics (14.5 and 5.2, respectively).

## Intentional Injuries

### Suicide

Native Americans are overrepresented in national estimates of alcohol-involved suicides. A CDC report (2009*a*) based on 2005–2006 data from the National Violent Death Reporting System presented findings on alcohol and suicide across ethnic groups. Recent alcohol use was reported among suicides in 46 percent of Native Americans, 30 percent of Hispanics, 26 percent of Whites, 16 percent of Blacks, and 15 percent of Asians. Among those tested for alcohol, the rates of intoxication (BAC higher than or equal to 0.08) were highest for Native Americans (37 percent), followed by Hispanics (29 percent), Whites (24 percent), Blacks (14 percent), and Asians (12 percent). Age-groups identified as being at high risk for alcohol-involved suicide included Native Americans ages 30 to 39 (54 percent of suicide victims had BACs higher than or equal to 0.08), Native Americans and Hispanics ages 20 to 29 (50 percent and 37 percent, respectively), and Asians ages 10 to 19 (29 percent). Males were at higher risk than female drinkers in all ethnic groups except Native Americans; the percentages of alcohol intoxication among Native American suicides were equal for males and females (37 percent).

### Violence

Ethnic groups are differentially affected by alcohol-attributed violence, including intimate-partner violence (IPV). Alcohol plays an important role in IPV and other types of relationship conflicts (Field and Caetano 2004; Leonard and Eiden 2007). Based on data from the National Study of Couples, general rates of male-to-female partner violence (MFPV) and female-to-male partner violence (FMPV), are highest among Black couples (23 percent and 30 percent, respectively), followed by Hispanic (17 percent and 21 percent) and White (12 percent and 16 percent) couples (Caetano et al. 2000). The National Study of Couples provides general population data on IPV, which includes mostly moderate violence and may differ from other studies of severe violence. In this study, regardless of ethnicity, men were more likely than women to report drinking during partner violence. Drinking during a violent episode by the male or the female partner, respectively, was more frequent among Blacks (MFPV: 41.4 percent and 23.6 percent; FMPV: 33.7 percent and 22.4 percent) than among Whites (MFPV: 29.4 percent and 11.4 percent; FMPV: 27.1 percent and 14.7 percent) and Hispanics (MFPV: 29.1 percent and 5.4 percent; FMPV: 28.4 percent and 3.8 percent). Longitudinal findings, using 5-year National Study of Couples data, identified female-partner alcohol problems (i.e., alcohol dependence symptoms and social problems) in Black couples and male- and female-partner alcohol consumption in White couples as risk factors for IPV (Field and Caetano 2003). Some evidence also suggests that interethnic couples, involving White, Black, and Hispanic partners of different ethnic backgrounds, are a high-risk group for relationship violence. Relative to intraethnic couples, these interethnic couples had higher prevalence rates of IPV, which was associated with binge drinking and alcohol problems among male partners (Chartier and Caetano 2012).

Alcohol also contributes to violence victimization among Native Americans (Yuan et al. 2006). Several studies indicate that Native Americans are at greater risk for alcohol-related trauma (e.g., IPV, rape, and assault) compared with other U.S. ethnic groups (Oetzel and Duran 2004; Wahab and Olson 2004). Based on 1992–2001 National Crime Victimization Survey data, the U.S. Department of Justice (Perry 2004) reported that 42 percent of all violent crimes (i.e., rape, sexual assault, robbery, aggravated assault, and simple assault) were committed by an offender who was under the influence of alcohol. In particular, Native American violent crime victims were more likely (62 percent) than other violent crime victims to report alcohol use by their offender, including Whites (43 percent), Blacks (35 percent), and Asians (33 percent).

## Fetal Alcohol Syndrome

Using data from the 2001–2002 NESARC, Caetano and colleagues (2006) examined alcohol consumption, binge drinking, and alcohol abuse and dependence among women who were pregnant during the past year. Most women (88 percent) who reported being pregnant and also a drinker at any point in the past 12 months indicated that they did not drink during pregnancy. Rates of past-year alcohol abuse (0.8 percent to 2.3 percent) and dependence (1.2 percent to 2.8 percent) were similar and low in White, Black, Hispanic, and Asian pregnant women. Binge drinking and alcohol consumption without binge drinking among pregnant women were highest in Whites (21.1 percent and 45.0 percent, respectively) compared with other ethnic groups (0 percent to 10.7 percent and 21.0 percent to 37.3 percent). White women in this study were at greater risk for an alcohol-exposed pregnancy. However, other studies found that Black, Hispanic, and Asian women were less likely to reduce or quit heavy drinking after becoming pregnant (Morris et al. 2008; Tenkku et al. 2009). Blacks and Native Americans are at greater risk than Whites for FAS and fetal alcohol spectrum disorders (Russo et al. 2004). From 1995 to 1997, FAS rates averaged 0.4 per 1,000 live births across data-collection sites for the Fetal Alcohol Syndrome Surveillance Network and were highest for Black (1.1 percent) and Native American (3.2 percent) populations (CDC 2002).

## Gastrointestinal Diseases

Liver disease is an often-cited example of the disproportionate effect of alcohol on health across ethnic groups. Native Americans have higher mortality rates for alcoholic liver disease than other U.S. ethnic groups (see figure). According to the National Vital Statistical Reports (Miniño et al. 2011) on 2008 U.S. deaths, age-adjusted death rates attributed to alcoholic liver disease for Native American men and women were 20.4 and 15.3 per 100,000 people, respectively, compared with 6.9 and 2.4 per 100,000 for men and women in the general population.

Blacks and Hispanics have greater risk for developing liver disease compared with Whites (Flores et al. 2008), and death rates attributed to alcohol-related cirrhosis across populations of Whites, Blacks, and Hispanics are highest for White Hispanic men (Yoon and Yi 2008). Blacks show a greater susceptibility than Whites to alcohol-related liver damage, with risk differences amplified at higher levels of consumption (Stranges et al. 2004). Based on data from the National Center for Health Statistics, 1991–1997, mortality rates for cirrhosis with mention of alcohol were higher in White Hispanics and Black non-Hispanics compared with White non-Hispanics (Stinson et al. 2001). Male mortality rates for alcohol-related cirrhosis in White Hispanics and non-Hispanic Blacks were 114 percent and 24 percent higher, respectively, than the overall male rate (5.9 deaths per 100,000 people); female rates in White Hispanics and non-Hispanic Blacks were 16 percent and 47 percent higher than the overall female rate (1.9 deaths per 100,000 people). In contrast, death rates for White non-Hispanic and Black Hispanic males and females were lower than overall rates for each gender. In addition, there is considerable variation in deaths from liver cirrhosis across Hispanic subgroups, with mortality rates highest in Puerto Ricans and Mexicans and lowest in Cubans (Yoon and Yi 2008).

## Cardiovascular Diseases

Although moderate alcohol consumption has been associated with a reduced risk for coronary heart disease (CHD) (Goldberg and Soleas 2001), there is some evidence that ethnic groups differ in terms of this protective effect, particularly for Blacks compared with Whites. Sempos and colleagues (2003) found no protective health effect for moderate drinking in Blacks for all-cause mortality, as previously reported in Whites. Kerr and colleagues (2011) reported the absence of this protective effect for all-cause mortality in Blacks and Hispanics. Similar findings have been described for hyper-tension and CHD risks in Black men compared with White men and women (Fuchs et al. 2001, 2004) and for mortality among Black women without hypertension (Freiberg et al. 2009). Mukamal and colleagues (2010) also showed that the protective effects of light and moderate drinking in cardiovascular mortality were stronger among Whites than non-Whites. Pletcher and colleagues (2005) found evidence that the dose-response relationship between alcohol consumption and increased coronary calcification, a marker for CHD, was strongest among Black men.

## Cancers

In 1988, the WHO International Agency for Research on Cancer (IARC) reviewed the epidemiologic evidence on the association between alcohol consumption and cancer and found a consistent association between alcohol consumption and increased risk for cancers of the oral cavity, pharynx, larynx, esophagus, and liver (IARC 1988). Regardless of ethnicity, the risk of developing these cancers is significantly higher among men than women (National Cancer Institute 2011*c*, *d*, *e*). The incidence and mortality rates for these cancers also vary across ethnic groups. Regarding cancers of the oral cavity and pharynx, incidence rates among White and Black men are comparable (16.1 and 15.6 per 100,000, respectively); however, mortality rates are higher among Black men (6.0 versus 3.7 per 100,000 for White men) (National Cancer Institute 2011*e*). For cancer of the larynx, both incidence and mortality rates are higher among Black men than among White men (incidence, 9.8 and 6.0; mortality, 4.4 and 2.0) (National Cancer Institute 2011*c*). Although these differences may be explained by differential use of alcohol and tobacco in relation to gender and ethnicity, there is some evidence that even after controlling for alcohol and tobacco use, Blacks continue to be at increased risk for squamous cell esophageal cancer and cancers of the oral cavity and pharynx (Brown et al. 1994; Day et al. 1993).

The majority (approximately 90 percent) of all primary liver cancers are hepatocellular carcinomas (HCC) (Altekruse et al. 2009). Alcohol-related and non–alcohol-related liver cirrhosis usually precede HCC and are the two most common risk factors (Altekruse et al. 2009; El-Serag 2011; Pelucchi et al. 2006). The relative risk for developing this cancer increases with increased levels of alcohol consumption (Pelucchi et al. 2006). By ethnic group, 2003–2005 age-adjusted incidence rates for HCC per 100,000 persons were highest among Asians (11.7), followed by Hispanics (8.0), Blacks (7.0), Native Americans (6.6), and Whites (3.9) (Altekruse et al. 2009). Death rates for HCC per 100,000 people also are higher among minority groups (i.e., 8.9, 6.7, 5.8, 4.9, and 3.5 for Asians, Hispanics, Blacks, Native Americans, and Whites, respectively).

In 2007, the IARC reconvened and added breast and colorectal cancers to the list of cancers related to alcohol use (Baan et al. 2007). Research has demonstrated consistent, albeit weak, dose-response relationships between alcohol consumption and these cancers (Cho et al. 2004; Collaborative Group on Hormonal Factors in Breast Cancer 2002; Moskal et al. 2007; Singletary and Gapstur 2001). Alcohol consumption also contributes to the stage at which breast cancer is diagnosed (Hebert et al. 1998; Trentham-Dietz et al. 2000; Vaeth and Satariano 1998; Weiss et al. 1996). This could be because of the timing of disease detection, since heavy drinking has been associated with a lack of mammography utilization (Cryer et al. 1999). Alcohol consumption also may contribute to more rapid tumor proliferation (Singletary and Gapstur 2001; Weiss et al. 1996). Data from the Surveillance, Epidemiology, and End Results (SEER) Program indicate that White women, relative to women from ethnic minority groups, have higher incidence rates of breast cancer (i.e., Whites, 127.3; Blacks, 119.9; Asians, 93.7; Native Americans, 92.1; and Hispanics, 77.9 per 100,000 people) (National Cancer Institute 2011*a*). Black women, however, are more likely to be diagnosed with advanced disease (Chlebowski et al. 2005) and have significantly higher mortality rates than White women (i.e., 32.0 per 100,000 versus 22.8 per 100,000 people) (Chlebowski et al. 2005; National Cancer Institute 2011*a*). Regarding colorectal cancer, Blacks have higher incidence (67.7) and mortality (51.2) rates than all ethnic groups combined (55.0 and 41.0, respectively) (National Cancer Institute 2011*b*). Unfortunately, little is known about how drinking differentially affects ethnic differences in breast and colorectal cancers.

## Diabetes

In 2010, the prevalence of diabetes was 7.1 percent, 12.6 percent, 11.8 percent, and 8.4 percent among Whites, Blacks, Hispanics, and Asians, respectively (National Institute of Diabetes and Digestive and Kidney Diseases 2011). Age-adjusted mortality rates in 2007 were 20.5, 42.8, 28.9, and 16.2 per 100,000 people among Whites, Blacks, Hispanics, and Asians (National Center for Health Statistics 2011). Data on mortality rates for diabetes among Hispanics may be underreported as a result of inconsistencies in the reporting of Hispanic origin on death certificates (Heron et al. 2009). Despite higher risks for the development of and death from diabetes in Hispanics and Blacks compared with Whites, little evidence is available to delineate the relationship of alcohol to diabetes across ethnic groups. Studies among both diabetics and nondiabetics demonstrate a J- or U-shaped curve between alcohol consumption and insulin sensitivity (Bell et al. 2000; Davies et al. 2002; Greenfield et al. 2003; Kroenke et al. 2003). Likewise, two large epidemiologic studies among diabetic subjects show that moderate alcohol consumption is associated with better glycemic control (Ahmed et al. 2008; Mackenzie et al. 2006). An important limitation of these studies, however, is that few included ethnic minority groups or failed to emphasize possible differences in relation to ethnicity in their analyses.

## Infectious Diseases

Among the infectious diseases attributable to alcohol (e.g., pneumonia, tuberculosis) (WHO 2011), human immunodeficiency virus (HIV) and acquired immunodeficiency syndrome (AIDS) are most relevant to U.S. ethnic health disparities. In 2009, Blacks represented 44 percent of new HIV infections and Hispanics represented 20 percent. Infection rates by gender for Blacks were 15 times (for men) and 6.5 times (for women) those of Whites, and rates for Hispanics were 4.5 times for men and 2.5 times for women, compared with rates for Whites (CDC 2011). In addition, alcohol consumption has been associated with increased HIV infection risk (Bryant et al. 2010). Caetano and Hines (1995) showed that heavy drinking predicted high-risk sexual behaviors in White, Black, and Hispanic men and women, with more Blacks than Whites and Hispanics reporting risky sexual behaviors. Among HIV-infected patients, there also is evidence that increased alcohol consumption negatively affects adherence to antiretroviral medication regimens (Chander et al. 2006; Cook et al. 2001; Samet et al. 2004) and HIV disease progression (Conigliaro et al. 2003; Samet et al. 2003). Despite these strong individual associations between ethnicity and HIV/AIDS and alcohol and HIV/AIDS, there is limited research across ethnicities on alcohol use and HIV infection or disease progression.

## Conclusions

This article identifies U.S. ethnic-group differences in alcohol-attributed social and health-related harms. Three minority ethnicities are particularly disadvantaged by alcohol-related harms. Native Americans, relative to other ethnic groups, have higher rates of alcohol-related motor vehicle fatalities, suicide, violence, FAS, and liver disease mortality. Unlike other ethnic groups, in which men are primarily at risk for alcohol-related harms, both Native American men and women are high-risk groups. Hispanics have higher rates of alcohol-related motor vehicle fatalities, suicide, and cirrhosis mortality. Blacks have higher rates of FAS, intimate partner violence, and some head and neck cancers, and there is limited empirical support in Blacks for a protective health effect from moderate drinking. These patterns of findings provide recognition of the health disparities in alcohol-attributed harms across U.S. ethnicities. However, further research is needed to identify the mechanisms that give rise to and sustain these disparities in order to develop prevention strategies. The contributing factors include the higher rates of consumption found in Native Americans and Hispanics, but more broadly range from biological factors to the social environment. More research on the relationship of alcohol to some cancers, diabetes, and HIV/AIDs across ethnic groups is also needed. There is limited evidence for how drinking differentially affects ethnic differences in breast and colorectal cancers and in diabetes and HIV/AIDS onset and care, and few findings for how alcohol-attributed harms vary across ethnic subgroups.

## Figures and Tables

**Figure:**
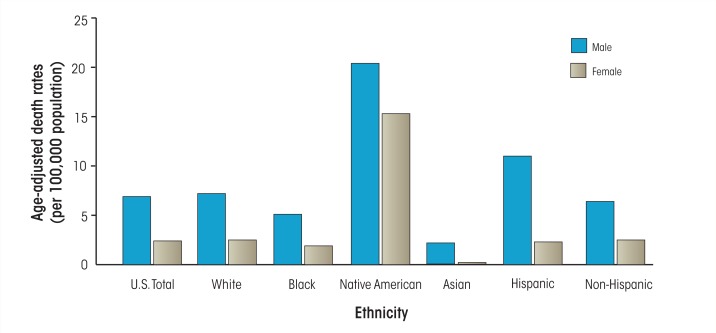
In 2008, age-adjusted death rates attributed to alcoholic liver disease for native american men and women were 20.4 and 15.3 per 100,000 people, respectively, compared with 6.9 and 2.4 for men and women in the general population. SOURCE: Miniño, A.M. et al., Deaths: Final data for 2008. *National Vital Statistics Reports* 59(10):1–52, 2011.
